# Lubricating Grease Thickness Classification of Steel Wire Rope Surface Based on GEMR-MobileViT

**DOI:** 10.3390/s25092738

**Published:** 2025-04-26

**Authors:** Ruqing Gong, Yuemin Wang, Fan Zhou, Binghui Tang

**Affiliations:** College of Power Engineering, Naval University of Engineering, Wuhan 430033, China; m22382401@nue.edu.cn (R.G.); zf422725587@163.com (F.Z.); tangbinghui0816@163.com (B.T.)

**Keywords:** image classification, deep learning, lightweight model, MobileViT, EMA, steel wire rope, grease, lubrication

## Abstract

Proper surface lubrication with optimal grease thickness is essential for extending steel wire rope service life. To achieve automated lubrication quality control and address challenges like variable lighting and motion blur that degrade recognition accuracy in practical settings, this paper proposes an improved lightweight GEMR-MobileViT. The model is designed to identify the grease thickness on steel wire rope surfaces while mitigating the high parameters and computational complexity of existing models. In this model, part of the standard convolution is replaced by GhostConv, a novel efficient multi-scale attention (EMA) module is introduced into the local expression part of the MobileViT block, and the structure of residual connections within the MobileViT block is designed. A transfer learning method is then employed. A custom dataset of steel wire rope lubrication images was constructed for model training. The experimental results demonstrated that GEMR-MobileViT achieved a recognition accuracy of 96.63% across five grease thickness categories, with 4.19 M params and 1.31 GFLOPs computational complexity. Compared to the pre-improvement version, recognition accuracy improved by 4.4%, while its parameters and computational complexity were reduced by 15.2% and 10.3%, respectively. When compared with current mainstream classification models such as ConvNeXtV2, EfficientNetV2, EdgeNeXt, NextViT, and MobileNetV4, our GEMR-MobileViT achieved superior recognition accuracy and demonstrated significant advantages in its model parameters, striking a good balance between recognition precision and model size. The proposed model facilitates deployment in steel wire rope lubrication working sites, enabling the real-time monitoring of surface grease thickness, thereby offering a novel approach for automating steel wire rope maintenance.

## 1. Introduction

**Figure 1:**
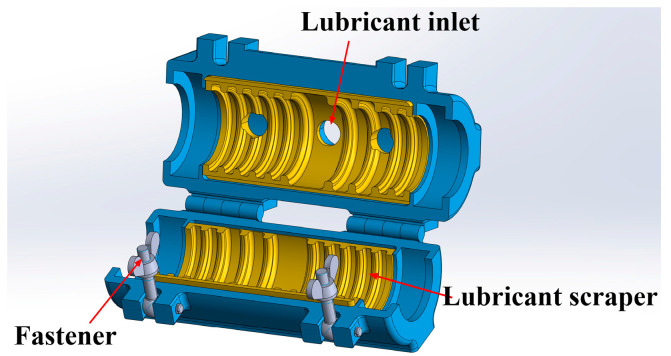
Structure diagram of wire rope lubricator.

Wire ropes are extensively utilized in engineering due to their high strength, good flexibility, and strong load-bearing capacity. Lubrication is an essential part of wire rope daily care and maintenance. Grease helps reduce wear, prevent corrosion, and slow the propagation of fatigue micro-cracks on the wire surface [1]. Lubricated wire ropes have a higher crack initiation threshold than unlubricated wire ropes [2]. Consequently, adequate and consistent lubrication can prolong the service lifespan of wire ropes by two to three times. Currently, specialized wire rope lubrication devices are commonly used in industrial settings, offering benefits such as high efficiency, improved control, and uniform lubrication [3,4,5]. The structure of the wire rope lubrication device is illustrated in Figure 1. The apparatus comprises a lubricating chamber, a grease scraper made of a resin liner, and other components. The wire rope passes through the lubricating apparatus at a consistent speed. A uniform grease film is formed on the surface through its relative motion with the scraper, thereby protecting the wire rope.

Steel wire ropes should have grease applied to their surface within a specific range in thickness. The grease layer will not effectively lubricate and protect against corrosion if it is excessively thin. On the other hand, an excessively thick coating could cause the grease to fling off while the machine is operating, contaminating the workspace. The grease thickness on the surface of steel wire ropes frequently varies with adjustments in the lubrication device’s parameters in real-world lubrication operations. For example, the scraper may experience wear from extended contact with the wire rope, which can increase the amount of grease on the wire rope surface. The amount of grease coating is also influenced by the relative motion speed between the lubrication device and the wire rope. Therefore, monitoring and controlling the grease thickness on the surface of steel wire ropes is a crucial step in the lubrication process. At present, the assessment of the lubrication status of steel wire rope surfaces primarily relies on manual inspection. When measuring the grease thickness on steel wire rope surfaces with traditional measurement tools, there are a number of difficulties: (1) the grease, being in a liquid or soft solid state, poses challenges for traditional measuring tools to achieve accurate measurements; (2) the surface of the steel wire rope is inherently uneven and irregular, with peaks and valleys between its strands, resulting in an uneven distribution of grease; and (3) the conditions in engineering field environments are frequently limited, making it difficult to deploy high-precision and expensive measuring devices. Therefore, it is crucial to find an efficient and cost-effective method for identifying the lubrication status on the surface of steel wire ropes.

There have been certain advancements in machine vision technology for identifying surface damages on steel wire ropes, such as the work by Zhou et al. [6], who implemented an improved YOLOv5 algorithm for detecting surface damages on moving mine steel wire ropes, achieving an average detection accuracy (mAP) of 82.3%. Jiang et al. [7] employed the Mask-RCNN network to segment images of steel wire ropes and determined the lay length of the rope from resulting Voronoi diagrams, yielding an average error of 1.0672 mm. Pan et al. [8] performed feature extraction on images of damaged steel wire ropes and employed the KNN method to categorize local defects, achieving a detection accuracy of 94.35% for failure categories. Nevertheless, none of this research has examined the application of imaging algorithms to ascertain the lubricating condition of steel wire ropes. The surface grease on steel wire ropes may become contaminated with tiny dust particles and impurities during operation, and reflections can arise under varying lighting circumstances, resulting in complex color details, substantial noise, and glare in the images. Moreover, capturing dynamic steel wire ropes might result in blurred images, complicating image characteristics and hindering feature extraction, which would lead to the suboptimal performance of traditional machine learning classification methods.

In recent years, with the rapid development of computer vision technology, the application of machine vision and deep learning in product inspection, defect detection, and quality control has become increasingly widespread. For example, Semitela et al. [9] achieved automation in the quality monitoring of coating surfaces for heating equipment by developing an automated defect detection and classification system based on ResNet50. Ficzere et al. [10] designed an algorithm utilizing machine vision and MATLAB 9.8.0.1721703 to measure the coating thickness of tablets, estimating the weight gain of coated tablets by visually comparing the diameter differences between uncoated and coated tablets. Gray-level co-occurrence matrix texture analysis and autoencoder classification techniques were used by Han et al. [11] to classify fuel injection nozzle surfaces to determine the presence or absence of coatings. They also employed a YOLO-CNN detection algorithm to locate coating interfaces, achieving an accuracy of over 95%. To detect the thickness of the retinal nerve fiber layer (RNFL), Mariottoni et al. [12] utilized a pretrained residual neural network, ResNet34, demonstrating excellent accuracy with a segmentation-free classification algorithm across both high-quality image sets and those containing errors or artifacts. Based on the aforementioned research, detection techniques that integrate deep learning and machine vision hold great potential for widespread application in sectors such as manufacturing and healthcare. Nevertheless, while the models utilized in these studies demonstrate high accuracy for their respective tasks, they do not take into account scenarios with limited hardware resources. The models above have relatively large number of parameters and substantial sizes; for example, the size of ResNet34 is approximately 80 MB, with around 21 M parameters.

Considering the diverse and complex operational environments of steel wire rope equipment, the proposed model should strive for high classification accuracy while being as lightweight as possible to accommodate resource-constrained deployment environments. Similar issues are faced in agricultural engineering; for example, to achieve the real-time recognition of plant diseases affecting crops such as wheat, coffee, and rice, Li et al. [13] proposed an improved PMVT model based on MobileViT, achieving an increase in accuracy ranging from 1% to 3.3% across three datasets. However, their model’s parameter size and FLOPs were 5.06 M and 1.59 GFLOPs, respectively, representing increases of 0.11 M and 0.13 GFLOPs compared to the original MobileViT. For plant leaf disease detection, Singh et al. [14] trained MobileViT with synthetic data produced by LeafyGAN, which improved accuracy by 20.7% on their PlantDoc dataset. However, they did not take into account the compression of their model, and the model’s parameters stayed the same. Therefore, based on the above research, this paper selects MobileViT as the basic model for the classification task of wire rope surface grease thickness and designs improvement strategies for both recognition accuracy enhancement and model compression. The goal is to improve model performance while also reducing hardware requirements.

In this research, we introduce a deep learning-based image classification approach to the field of steel wire rope maintenance engineering, selecting the lightweight MobileViT as the base network model due to its low computational cost and fast inference speed. We first reduce the model’s number of parameters and computational complexity by replacing some standard convolutions with the lightweight GhostConv. Next, to address the challenges of identifying images of lubricated steel wire ropes under varying lighting conditions and dynamic scenes, we design an improved MobileViT block that combines an efficient multi-scale attention module (EMA) and residual connections. Finally, the transfer learning method is used to further improve image recognition accuracy and speed up training. We refer to this improved model as GEMR-MobileViT. Experimental results on our self-constructed steel wire rope image dataset demonstrate that our GEMR-MobileViT model outperforms the current mainstream image classification models, and possesses significant advantages in terms of model lightweighting, making it suitable for deployment in embedded or mobile devices. This research facilitates the real-time monitoring of grease thickness on moving steel wire ropes under various working conditions. The main contributions of this paper are as follows:

- An image acquisition platform was established, and steel wire rope lubrication experiments were conducted using a self-made lubrication device. A total of 1874 images of the lubricated surface of steel wire ropes were collected, and through data augmentation, the image dataset was expanded to 3478 images, which were categorized into five classes based on the thickness of the grease on the wire rope surface.
- An improved GEMR-MobileViT network was proposed, incorporating a lightweight GhostConv and EMA module, combined with residual connections to enhance the model’s capability for feature extraction and fusion in images. Transfer learning techniques were employed to improve the model’s training efficiency.
- The impact of adding the EMA module at different positions within the network was explored, and a comparison was made between the effects of common attention mechanism modules (SE, CA, CBAM) and EMA.
- Comparative performance experiments were conducted on the self-constructed steel wire rope image dataset, evaluating the GEMR-MobileViT model against other mainstream image classification models (ConvNeXt, EfficientNet, Swin Transformer, ViT, ResNet, GoogLeNet).

## 2. Data Collection and Preprocessing

### 2.1. Steel Wire Rope Lubrication Experiment and Image Acquisition

**Figure 2:**
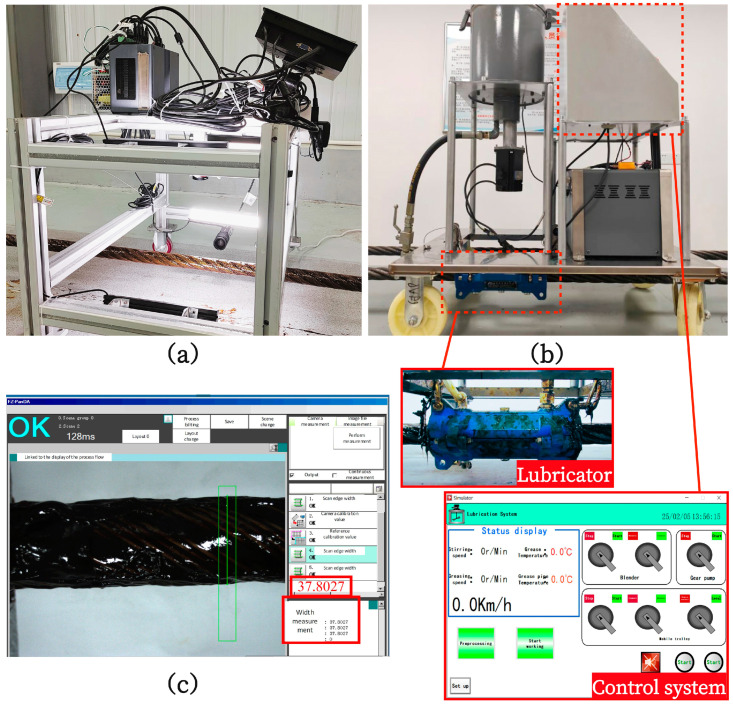
(**a**) Image acquisition equipment, (**b**) lubricating device, and (**c**) visual measurement interface.

The experimental setup used for image acquisition is shown in Figure 2a and includes the following: (1) four bar light sources positioned at different locations; (2) two 20-megapixel GigE monochrome area scan cameras (MV-CS200-10GC, Hikvision Digital Technology Co., Ltd., Hangzhou, China) paired with two C-mount lenses (MVL-KF1640-25MP, Hikvision Digital Technology Co., Ltd., Hangzhou, China) with a focal length of 16 mm, capable of capturing images at a resolution of 5472 × 3647; and (3) an industrial PC with data storage and light source control capabilities. The two sets of camera lenses are fixed on specially designed adjustable brackets, allowing for the continuous acquisition of images of the steel wire rope from different angles and under varying lighting conditions by adjusting the camera position and light source brightness.

The wire rope lubrication experiment was conducted using a custom-built wire rope greasing device. The automated lubrication system comprises an electrical control system, a grease heating and pumping system, and an applicator mechanism, with the complete assembly illustrated in Figure 2b. The control system enables the adjustment of the grease pump motor speed and the precise setting of the grease heating temperature. Additionally, the lubricator accommodates interchangeable scraping liners with varying diameters. By systematically modifying these device parameters while simultaneously adjusting the moving speed of the lubrication apparatus along the wire rope across multiple experimental trials, the thickness of the lubricant coating applied on the wire rope surface can be effectively controlled.

The subjects of the experiment were a specific type of special steel wire rope with a nominal diameter of φ = 365 mm. A certain type of special grease was used for lubrication. Greasing experiments were conducted on five wire rope segments, each with a length L = 7 m. Segments 1 to 4 were used to collect images for the training and validation sets, while Segment 5 served as an independent test set. This segregation ensured no overlap between the test and training data, preventing data leakage while validating the model’s generalization capability in real-world scenarios.

To accurately classify the grease thickness on wire rope surfaces, we employed a high-precision vision measurement system to evaluate diameter changes before and after greasing (Figure 2c). The system captures the distance between the rope’s left and right edges with a measurement accuracy of ±0.02 mm and repeatability error within 1.5%. By calculating diameter differences pre- and post-greasing, we derived the actual grease thickness and classified it into five categories: ungreased, thin, proper, thick, and excessive. The specific thickness ranges for each category are detailed in Table 1.

**Figure 3:**
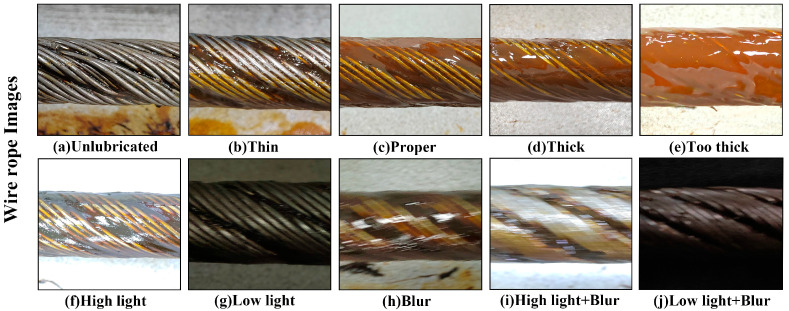
(**a**–**e**) Five categories of wire rope surface grease thickness. (**f**–**j**) Images under different lighting conditions and clarity levels.

Using our custom-built image acquisition equipment, we captured surface images of wire ropes across all five grease thickness categories, obtaining a total of 758 images. The collected images encompass various lighting conditions and clarity levels to simulate the diverse scenarios encountered in practical applications. Representative examples of the wire rope images corresponding to the five grease thickness categories under different conditions are presented in Figure 3.

### 2.2. Data Augmentation

The collected 758 original images were uniformly cropped to focus on the wire rope regions and proportionally segmented, yielding a total of 1874 images with standardized dimensions of 1300 × 1200 pixels. Among these, 193 images were independently collected as the test set from wire rope Segment 5. The remaining 1681 images obtained from Segments 1–4 were randomly divided into a training (1496 images) and a validation set (185 images) in an 8:1 ratio, resulting in an overall 8:1:1 distribution across the training, validation, and test sets.

To address any potential classification accuracy degradation caused by class imbalance in the training set, we implemented multiple data augmentation techniques. These techniques could effectively mitigate model bias toward specific classes and reduced overfitting risks [15]. The augmentation methods included rotation, scaling, translation, motion blur, Gaussian blur, grid distortion, adaptive histogram equalization, random brightness adjustments, and mask occlusion. Notably, augmentation was exclusively applied to the training set, expanding it from 1496 to 3100 images while maintaining approximate class balance (≈700 images per category). The final augmented dataset comprised 3478 images, with detailed class distributions provided in Table 2.

## 3. Classification Method of Wire Rope Surface Grease Thickness Based on GEMR-MobileViT

### 3.1. Basic MobileViT Model

**Figure 4:**
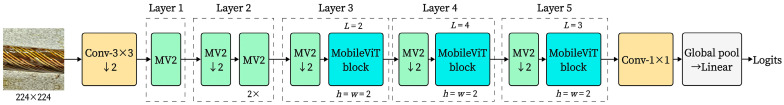
MobileViT network structure.

MobileViT, proposed in 2021, is a lightweight model that integrates Convolutional Neural Networks (CNNs) with the Vision Transformer architecture, designed for image classification and other computer vision tasks [16]. MobileViT combines the spatial inductive bias of CNNs with the global feature processing advantages of Vision Transformers, allowing the model to maintain a high performance while being computationally efficient and lightweight, making it compatible with deployment on mobile platforms and embedded devices. Considering the requirements for classification accuracy and ease of deployment, this paper selects MobileViT as the model for classifying the thickness of grease on steel wire rope surfaces. MobileViT is mainly composed of an MV2 module and MobileViT block. Images first pass through a 3 × 3 convolutional layer for local feature extraction, followed by a 1 × 1 convolutional layer for channel adjustment, then undergo continuous downsampling and pooling through multiple cascaded MV2 modules and MobileViT blocks for feature extraction, as illustrated in Figure 4.

**Figure 5:**
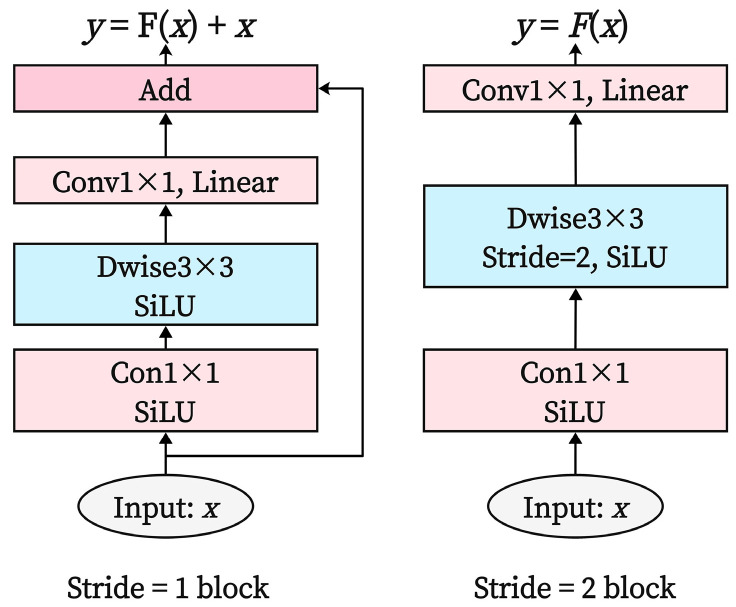
MV2 module structure.

**Figure 6:**
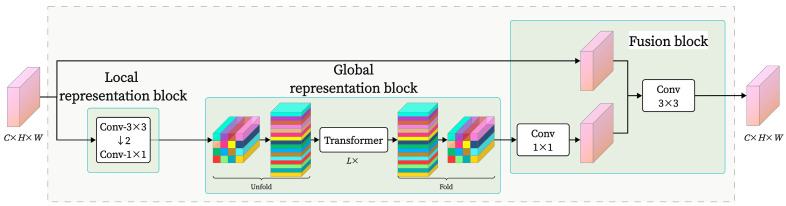
MobileViT block structure.

MV2 represents the MobileNetV2 module, as shown in Figure 5. The MobileNetV2 module efficiently extracts local features while reducing the model’s parameter count by integrating depthwise separable convolutions, inverted residual structures, and linear bottleneck techniques. The MobileViT block module is the core of MobileViT, as illustrated in Figure 6. It consists of three parts: local feature representation, global feature representation, and feature fusion. The global feature part incorporates a transformer module that establishes relationships between tokens through a Multi-Head Attention (MHA) mechanism, computing attention distributions for each position and capturing the global relationships between image patches, thereby enhancing the model’s focus on global information.

### 3.2. Improved GEMR-MobileViT Model

We present GEMR-MobileViT, an improved model that is suitable for classifying the thickness of grease on steel wire rope surfaces, based on MobileViT. For the core of MobileViT, namely the MobileViT block, we made three improvements: First, we replaced the standard convolution in the MobileViT block with GhostConv to achieve a lightweight network design, reducing the model’s parameters and computational complexity. Second, we introduced the EMA module before the transformer to enhance the model’s attention to important regions under different lighting and complex scenes, thereby improving the image classification feature fusion capability. Additionally, we designed residual connections to alleviate issues associated with the vanishing gradient problem and enhance the model’s utilization of input features. Finally, we employed transfer learning methods to further improve the model’s accuracy and accelerate training speed. The main improvements are shown in the following subsections.

#### 3.2.1. GhostConv

**Figure 7:**
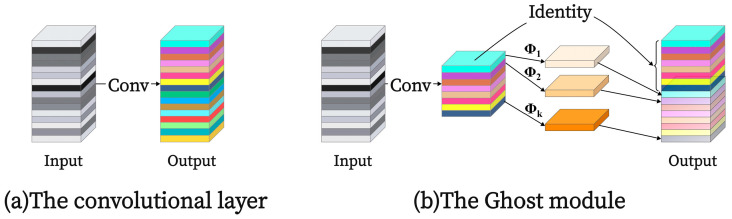
Comparison of regular convolution and GhostConv structure.

**Figure 8:**
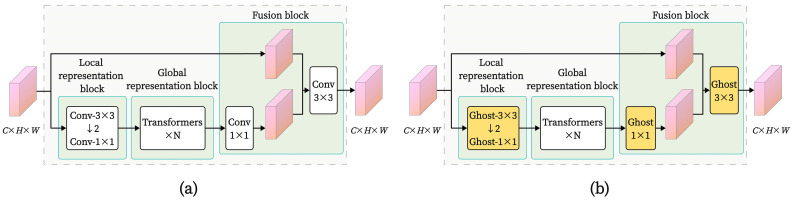
(**a**) MobileViT block; (**b**) MobileViT block replacing regular convolution with GhostConv.

GhostConv is derived from the GhostNet model proposed by Huawei Noah’s Ark Laboratory [17,18]. The core of GhostNet is to use the lightweight GhostConv to reduce the model parameters and calculation of convolution so as to optimize model parameters and computational complexity while maintaining model accuracy. The structure comparison of regular convolution with GhostConv is shown in Figure 7. GhostConv divides the convolution operation into two steps: First, it employs a small number of conventional convolution kernels to extract features from the input feature map. Second, it applies a linear transformation operation Φ on the generated feature map through deep convolution and finally obtains the final feature map through a splicing operation. The output feature of Ghost convolution is calculated as follows:

(1) $$Y^{\prime }=X*W_{p}$$

(2) $$Y=Cat(Y^{\prime },X*W_{c})$$

where *X* is the input features, *Y*′ is the ghost features, and *Y* is the final output features. *W_p_* and *W_e_* refer to the parameters of the standard convolution and the cheap operation, respectively. “Cat” indicates the concatenation operation. By replacing the standard convolution in the MobileViT block with lightweight GhostConv, the parameters and computational complexity of the MobileViT network are reduced, providing favorable conditions for deploying the model on-site in steel wire rope equipment. A comparison between the original MobileViT block and the MobileViT block with GhostConv replacing the standard convolution is illustrated in Figure 8.

#### 3.2.2. Efficient Multi-Scale Attention (EMA) Module

**Figure 9:**
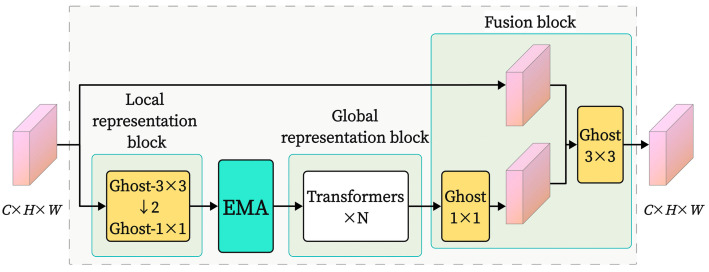
MobileViT block structure with EMA module added.

Motion blur and lighting variations add to the intricacy of image features in the steel wire rope field image dataset. With this complexity, it is difficult for current image classification models to use important feature information efficiently and achieve high classification accuracy when determining the grease thickness on steel wire rope surfaces. To address these issues, we designed an improved MobileViT model integrating an efficient multi-scale attention (EMA) module. Unlike conventional enhancements where the EMA is added independently to a certain layer of the network, we ingeniously embedded the EMA within the core component of the MobileViT model—the MobileViT block—as shown in Figure 9. By appending the EMA module after the local feature representation portion of the MobileViT block in Layer 3, it forms a cascaded relationship with the transformer encoder. This integration fully leverages the attention mechanism to enhance the model’s ability to classify complex images.

**Figure 10:**
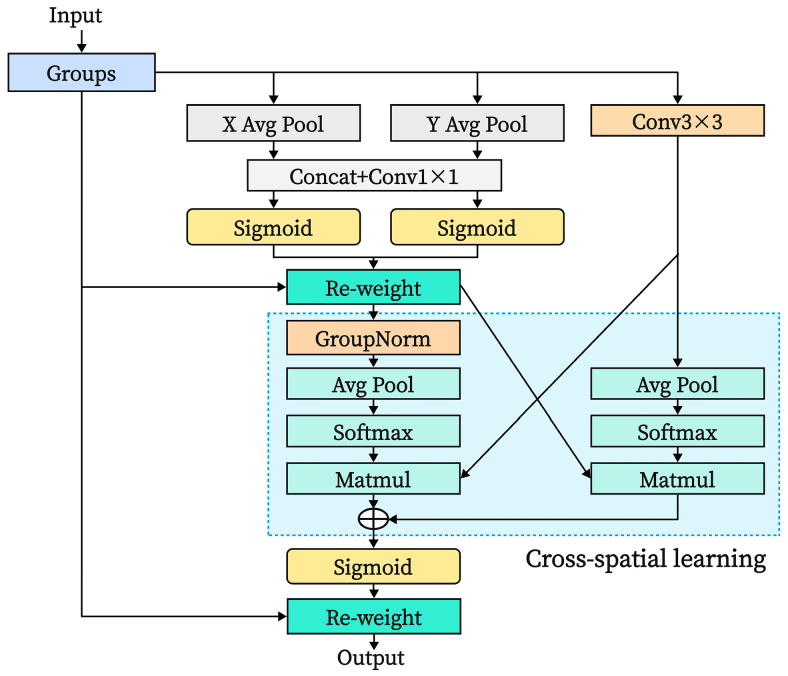
Efficient multi-scale attention (EMA) module structure.

The efficient multi-scale attention (EMA) module [19] differs from the sequential processing method of Coordinate Attention (CA) by using grouping and multi-scale structures for feature processing across different spatial dimensions. This method reduces computational costs while retaining the original channel dimensions, enabling the model to achieve enhanced pixel-level attention on high-level feature maps. In the EMA module, some of the channels are reshaped into the batch dimension and divided into multiple sub-features, allowing spatial semantic features to be evenly distributed across each feature group. The EMA mechanism is depicted in Figure 10, showing three parallel pathways for input feature extraction: two 1 × 1 paths and one 3 × 3 path. Within the 1 × 1 branches, two global average pooling operations are performed, and the channel encodings from two spatial directions are concatenated. The re-weight module aggregates these paths to enable cross-channel feature interaction. The 3 × 3 path captures multi-scale features using a 3 × 3 convolution and performs two-dimensional average pooling for channel encoding. Furthermore, EMA introduces a branch between the aggregated 1 × 1 and 3 × 3 paths, employing a cross-spatial learning strategy for global spatial information encoding. This achieves the fusion and rapid response of information features across channel and multi-scale spatial dimensions. The formula for the two-dimensional global pooling operation is

(3) $$AvgPool=\frac{1}{H\times W}\sum_{j}^{H}\sum_{i}^{W}X_{\partial }\left(i,j\right)$$

where *H* and *W* represent the height and width of the input feature map and $X_{\partial }\left(i,j\right)$ represents the pixel value in the *∂*-th channel of the feature map.

By integrating the EMA module after the local feature representation section of the MobileViT block, a cascading relationship is formed between the EMA mechanism and the transformer encoder. The EMA module first enhances local features, capturing fine-grained spatial information and contextual relationships. The transformer then applies additional global feature modeling to the improved features to capture long-range dependencies. The multi-level attention mechanism composed of this improvement enables the model to capture and integrate fine-grained features and global features in images at different levels, effectively enhancing the robustness and generalization ability of feature representation, thereby improving the accuracy of image recognition with different brightness and blur degrees.

#### 3.2.3. Residual Connections

The core idea of residual connections is to introduce a link between the input and output signals of a network layer, allowing input signals to be directly transmitted to deeper layers of the network without transformation, rather than being represented solely by the results of layer-by-layer forward propagation [20]. The output of a residual module is obtained by adding the original input signal to the output signal after it has undergone a function transformation. The calculation formula for residual connections is as follows:

(4) $$y=F\left(x,\left\{W_{i}\right\}\right)+x$$

where *x* is the input signal, *y* is the final output signal, and $F\left(x,\left\{W_{i}\right\}\right)$ represents the residual mapping to be learned.

**Figure 11:**
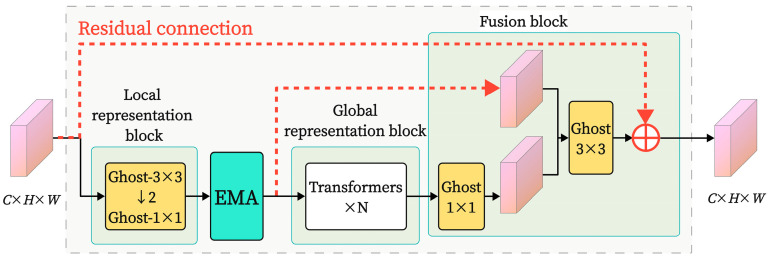
MobileViT block structure with added residual connections.

The residual connection structure helps to address the vanishing gradient problem in deep networks, facilitating the construction and optimization of more stable deep network structures. Models such as ResNet, DenseNet, and MobileViTv3 have improved their architectural design by applying residual connections [21]. Furthermore, residual connections also assist in feature reuse, enhancing the importance of shallow features in deeper network layers, thereby improving the model’s classification performance and generalization capability. Thus, as illustrated in Figure 11, two residual connections were added to the MobileViT block in response to the addition of the EMA module. The first residual connection links the overall input and output signals of the MobileViT block, while the second links the output signal of the EMA module with the input signal of the feature fusion part. The EMA’s feature extraction capability can be more effectively employed in the enhanced network layers by creating these residual connections, which allow the features it processes to directly participate in later computations while maintaining the effects of the attention mechanism.

#### 3.2.4. Transfer Learning

Transfer learning leverages features or knowledge a model has learned on a source task by using the similarities between training tasks to provide a reference for the target task. This method lowers the quantity of computational resources and training data needed to complete the target task. Through transfer learning methods, it is possible to use well-trained data from the source task to tackle learning challenges such as small samples and imbalanced datasets [22]. Transfer learning methods are categorized into sample-based, feature-based, parameter-based, and relation-based transfers. Due to the structural generality present in image classification tasks, pretrained weights on a larger dataset *D*_s_ can be transferred to a smaller dataset *D*_t_. By employing a fine-tuning approach for the target task training, the model’s parameters, either partially or fully, can be adjusted, thereby enhancing training efficiency and model performance.

However, due to the lack of datasets similar to lubricated steel wire ropes, it is challenging to adopt transfer learning methods that learn similar features from other datasets. Inspired by the research of Hitelman et al. [23], we employed a transfer learning approach that involves grouping the datasets so they can learn from each other. The steel wire rope image dataset was divided into two groups with an equal number of samples. The model was first trained on one group, and then the resulting model weights were used as pretrained weights to retrain on the other group. The learning rate was decreased during relearning to prevent overfitting. This interactive learning was carried out twice, and finally, the model was evaluated on the same test set. The model was able to learn the image features of the other portion of the dataset more rapidly by employing this transfer learning technique, which improved classification accuracy while cutting down on training time.

## 4. Results

### 4.1. Experimental Environment and Training Parameter Configuration

The experimental hardware environment consists of a 64-bit Windows 10 operating system, utilizing the PyTorch 2.4.1 deep learning framework, with a CUDA version of 12.1. All experimental models use the NVIDIA GeForce RTX 4070 SUPER GPU, and the CPU is a 13th Gen Intel(R) Core(TM) i5-13600KF @ 5.10 GHz equipped with 32 GB of RAM and operating within a Python 3.9.2 environment. The input image size for the model is 224 × 224. After experimental comparison, it was determined that the following training parameters are optimal: an initial learning rate set at 0.0001, a batch size of 16, and the Adam optimizer; the loss function is the cross-entropy loss function, and the learning rate adjustment strategy follows the cosine annealing algorithm; and the number of training epochs is set to 50.

### 4.2. Evaluation Metrics

To comprehensively evaluate the recognition performance of the model, we use accuracy and the F1-score as metrics for assessing the model’s performance. The model’s lightweight characteristics and inference speed are evaluated using model parameters (Params), floating-point operations per second (FLOPs), model size, and frames per second (*FPS*). The formulas for calculating these metrics are as follows:

(5) $$\mathrm{Accuracy}=\frac{TP+TN}{TP+TN+FP+FN}$$

(6) $$F1=2\times \frac{\mathrm{Precsion}\times \mathrm{Recall}}{\mathrm{Precsion}+\mathrm{Recall}}$$

(7) $$FPS=\frac{1000}{T_{pre}+T_{inf}+T_{post}}$$

where *TP* represents the number of true positive samples, *FP* denotes the number of false positive samples, *FN* indicates the number of false negative samples, and *TN* is the number of true negative samples. The unit of 1000 is in milliseconds (ms), with *T_pre_* representing the model’s preprocessing time, *T_inf_* as the model’s inference time, and *T_post_* as the model’s post-processing time. All model training in this paper was conducted through five independent experimental trials, with the final performance metrics calculated as the mean values across these five runs.

### 4.3. Results and Analysis

#### 4.3.1. Baseline Model Selection

**Figure 12:**
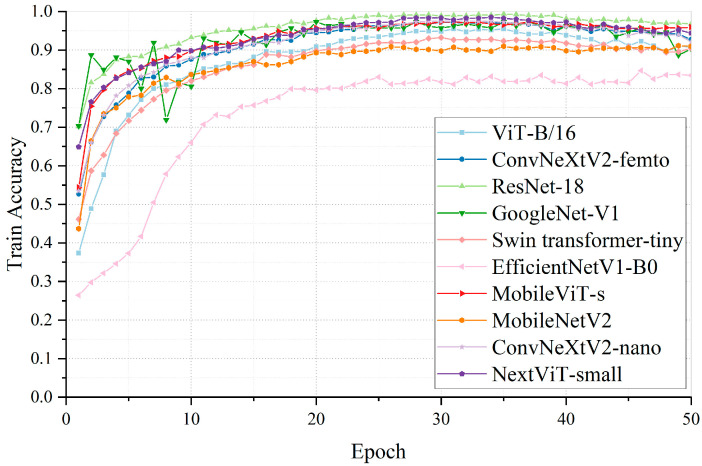
Training accuracy curves of different models.

To select the most suitable model for the wire rope surface grease thickness classification task, we first experimentally compared various mainstream deep-learning image classification models in recent years on a self-built wire rope image dataset. These models encompassed classic heavyweight architectures, state-of-the-art lightweight models, the Vision Transformer (ViT), and their hybrid architecture variants. For the lightweight models, multiple architecture versions with comparable parameter scales were selected to assess performance discrepancies among the different models under similar parameter conditions. The specific architectures, performance metrics, and parameters of each model are detailed in Table 3, and the accuracy variation curves during training are shown in Figure 12.

As demonstrated by the results, MobileViT-s achieves the optimal balance between classification accuracy and lightweight performance. Among the lightweight models, it attains the highest classification accuracy. When compared with heavier models, MobileViT-s demonstrates distinct advantages in model parameter volume and computational complexity, while also showcasing superior inference speed. After comprehensive consideration of the model’s classification accuracy and lightweight characteristics, we selected MobileViT-s as the backbone network and proceeded with improvements based on this architecture.

#### 4.3.2. Ablation Study

GEMR-MobileViT incorporates three structural optimizations based on MobileViT: (a) replacing the conventional convolutions in the MobileViT block with lightweight GhostConv; (b) introducing EMA into the local expression part of the MobileViT block; and (c) designing residual connections within the MobileViT block. To validate the effectiveness of these three improvements, ablation experiments were conducted based on our self-constructed wire rope image dataset. The results of the ablation experiments are shown in Table 4. In this table, model 1 is the original MobileViT, model 2 is MobileViT + GhostConv, model 3 is MobileViT + EMA, model 4 is MobileViT + residual connections, model 5 is MobileViT + GhostConv + EMA, model 6 is MobileViT + GhostConv + residual connections, model 7 is MobileViT + EMA + residual connections, and model 8 is MobileViT + GhostConv + EMA + residual connections.

The results show that model 2, which replaces conventional convolutions with GhostConv, achieves a reduction in model parameters and computational complexity by 0.76 M and 0.17 G FLOPs, respectively, compared to the original model, while maintaining nearly the same classification performance. This indicates that GhostConv can effectively eliminate redundant information in the model, reducing the overall number of parameters and computational complexity without affecting performance. Model 3, with the introduction of the EMA module, shows a minimal increase in computational complexity by 0.02 G FLOPs, with the model parameters remaining nearly unchanged, while the accuracy and F1-score improve by 2.59% and 2.31%, respectively. This suggests that the inclusion of the EMA module significantly enhances classification performance with only a slight increase in computational complexity. Comparing models 3, 4, and 7, model 4 does not show a significant performance improvement when only residual connections are added. However, model 7, which combines the EMA module and residual connections, demonstrates a clear performance improvement over models 3 and 4. This indicates that the combination of the EMA module and residual connections provides a positive synergistic effect, ensuring the effective transmission of output features from the EMA module between layers, allowing for thorough interaction between local and global feature information, enhancing the model’s feature fusion capability, and successfully increasing classification accuracy to 95.34%. Model 8 shows that GEMR-MobileViT with all three enhancements improves the accuracy and F1-score by 4.14% and 4.02%, respectively, compared to the original model. Furthermore, the model parameters and computational complexity are decreased by 15.2% and 10.3%, respectively, while the real-time inference speed (*FPS*) increases by 2.5 f·S^−1^ and the model size stays relatively constant. This indicates that GEMR-MobileViT achieves higher classification accuracy for wire rope images while maintaining lightweight model parameters and computational complexity.

#### 4.3.3. Transfer Learning Effects

The wire rope image dataset was randomly divided into two equal groups (Group 1 and Group 2). In Method 1, the model was trained on Group 1 and then retrained on Group 2. In Method 2, the model was trained on Group 2 and then retrained on Group 1. The learning rate for the second round of training was set to 2 × 10^−5^. In this study, each method was subjected to five experimental runs, and the mean value of these experiments was calculated. Ultimately, the average classification accuracy obtained from the two learning approaches was compared with the results from models that did not utilize transfer learning, as shown in Table 5. The comparison indicates that the model subjected to transfer learning achieved improvements of 0.26% in accuracy and 0.04% in F1-score, while the training time was reduced by 37%. This demonstrates that through transfer learning between different data groups, the model is able to make more effective use of limited data resources, significantly reduce training time, and enhance classification accuracy.

#### 4.3.4. Comparison of Attention Mechanisms

The MobileViT block is divided into three parts: local representation, global representation, and feature fusion. The transformer is located in the global representation part. To investigate the impact of adding the EMA module at different positions within the network, experiments were conducted by adding the EMA module after the local representation part and after the global representation part of the MobileViT block, respectively. The results are shown in Table 6.

The comparison indicates that the scores for all metrics are higher when the EMA module is added to the local representation position than when it is added to the global representation position. Specifically, the accuracy is 3.62% higher and the F1-score is 4.27% higher. This suggests that adding the EMA module before the transformer module in the local representation position yields a better model performance. The EMA can enhance the multi-scale perception capability and cross-spatial feature fusion ability of local feature extraction, thereby capturing detailed features more effectively. These features are then expressed globally by the transformer, resulting in an effective attention sequence cascade mechanism.

To verify the impact of the EMA module on the model, comparative experiments were conducted with the EMA and other commonly used lightweight attention modules in deep learning, such as SE, CA, and CBAM [24,25,26]. The SE module is a channel attention mechanism that adaptively adjusts channel weights. The CA module improves upon the SE module by introducing coordinate information to account for the correlation between channels and positions. The CBAM module includes attention mechanisms in both channel and spatial dimensions. The results of the comparison experiments are shown in Table 7; it shows that among the four commonly used lightweight attention modules, the EMA module exhibits the best overall performance in all aspects. This indicates that the choice to improve the model using EMA is reasonable and effective, as the addition of an efficient multi-scale attention mechanism enhances the model’s ability to extract features from complex scene images, enabling the model to focus more on key feature areas.

#### 4.3.5. Comparison of Different Models

To comprehensively evaluate and validate the classification performance of the GEMR-MobileViT model, a diverse set of widely recognized image classification network models were selected and trained on a self-built steel wire rope image dataset for result comparison. This ensemble included classical architectures such as GoogLeNet, ResNet, and ViT; state-of-the-art lightweight models like MobileViT, MobileViTv3, MobileNetV2, MobileNetV4, EfficientNet, ConvNeXtV1, ConvNeXtV2, and EdgeNeXt; and hybrid architecture variants of Vision Transformers (ViT) such as the Swin Transformer and NextViT. The results are presented in Table 8.

This shows that GEMR-MobileViT demonstrates a notable improvement over the baseline MobileViT-s, achieving an increase in both recognition accuracy and F1-score by 4.4% and 4.06%, respectively. Concurrently, it reduces the number of parameters and computational complexity by 15.2% and 10.3%, respectively. The increase in model size is minimal, at only 0.09 MB, while the real-time inference speed is enhanced by 2.79 f·S^−1^. GEMR-MobileViT demonstrates significant performance advantages in classification tasks, with both accuracy and F1-score surpassing all comparative models, including the lightweight MobileViTv3-s and the heavyweight NextViT-small. In terms of lightweight metrics, this model has a parameter count of only 4.2 M and a computational complexity of 1.31 G FLOPs, as well as an 18.99 MB model size—reductions of 86.32%, 77.37%, and 83.83%, respectively, compared to the similarly high-accuracy NextViT-small model—while maintaining a high inference speed of 112.05 FPS, outperforming most models of comparable scale.

Notably, MobileViTv3 represents the official third-generation improved version of MobileViT, incorporating modifications such as depthwise separable convolutions and revised feature fusion mechanisms. Our GEMR-MobileViT, developed by enhancing the MobileViT v1 architecture, exhibits a more efficient performance on the target task than MobileViTv3: it achieves a higher classification accuracy while maintaining nearly identical parameter counts and model size, thus achieving a more balanced performance profile. Additionally, compared to the conventional MobileNetV2, GEMR-MobileViT achieves a 6.47% accuracy improvement with a modest increase in its parameters and model size, with an inference speed (FPS) slightly lower than MobileNetV2, highlighting the effectiveness of its structural innovations.

**Figure 13:**
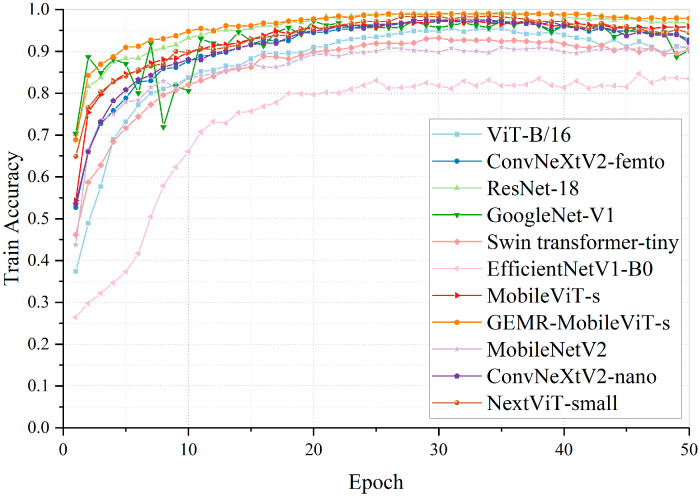
Training accuracy curves of GEMR-MobileViT and other models.

The progression of classification accuracy across the training epochs for each model is depicted in Figure 13. All models exhibit a trend towards stabilization after approximately 30 epochs, and GEMR-MobileViT achieves both the highest accuracy and fastest convergence, with relatively stable performance fluctuations. This suggests that GEMR-MobileViT offers significant advantages in the classification task for the thickness of lubricating grease on steel wire rope surfaces.

#### 4.3.6. Classification Performance of GEMR-MobileViT on Grease Thickness Images of Steel Wire Ropes

**Figure 14:**
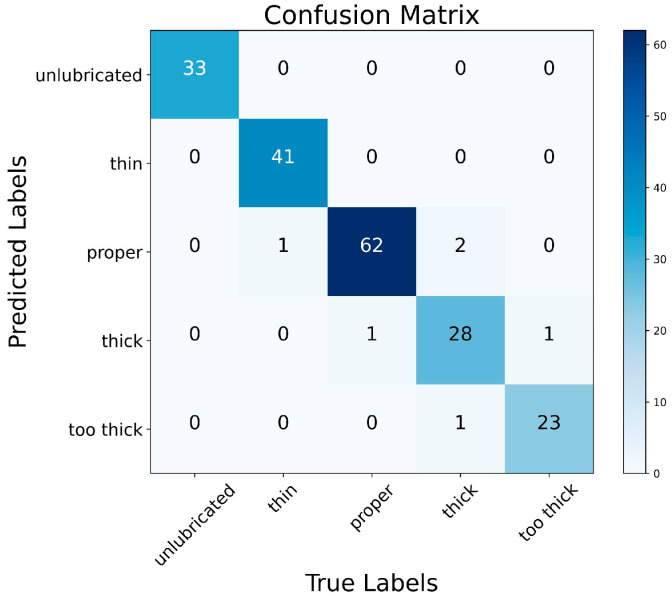
Confusion matrix.

The performance of GEMR-MobileViT in classifying the thickness of grease on wire rope surfaces is evaluated using a confusion matrix, as depicted in Figure 14. The results indicate that the model achieves relatively high recognition accuracy across the five thickness categories. And misclassifications predominantly occur between adjacent categories, such as between “thin” and “proper”, “proper” and “thick”, or “thick” and “too thick”. This indicates limited model sensitivity to fine thickness variations, highlighting the challenge of fine-grained classification in this domain.

**Figure 15:**
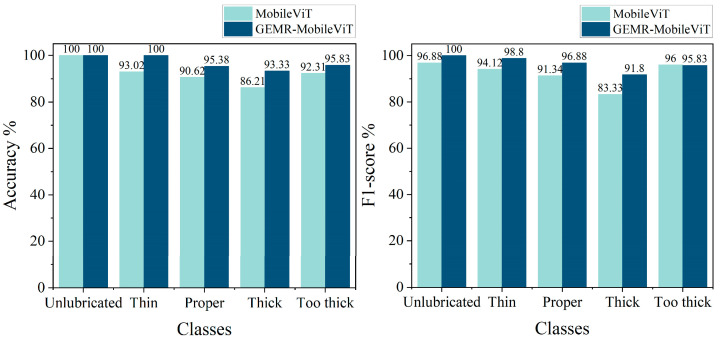
Classification accuracy and F1-score for different wire rope surface grease thicknesses.

**Figure 16:**
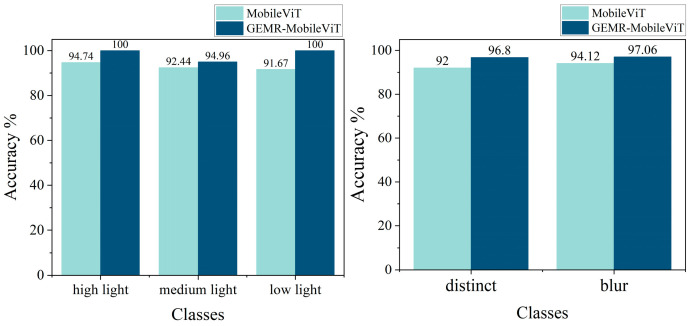
Classification accuracy under various lighting and clarity conditions.

Figure 15 presents the recognition accuracy and F1-score for the five thickness categories before and after the improvements to GEMR-MobileViT. The improved model demonstrates a significant enhancement in both its accuracy and F1-score across all categories, indicating the effectiveness of the proposed modifications. To further investigate the model’s robustness under varying environmental conditions, the test set images were categorized based on lighting conditions and clarity levels. The recognition accuracy under different scenarios is illustrated in Figure 16. Specifically, the improved model achieves a 5.26%, 2.52%, and 8.33% increase in accuracy under high, medium, and low lighting conditions, respectively. Additionally, recognition accuracy improves by 4.8% and 2.94% under clear and blurry shooting scenarios, respectively. These results demonstrate that our modifications significantly improve the fine-grained recognition ability and feature extraction capability of the model, particularly in complex and challenging scenes.

**Figure 17:**
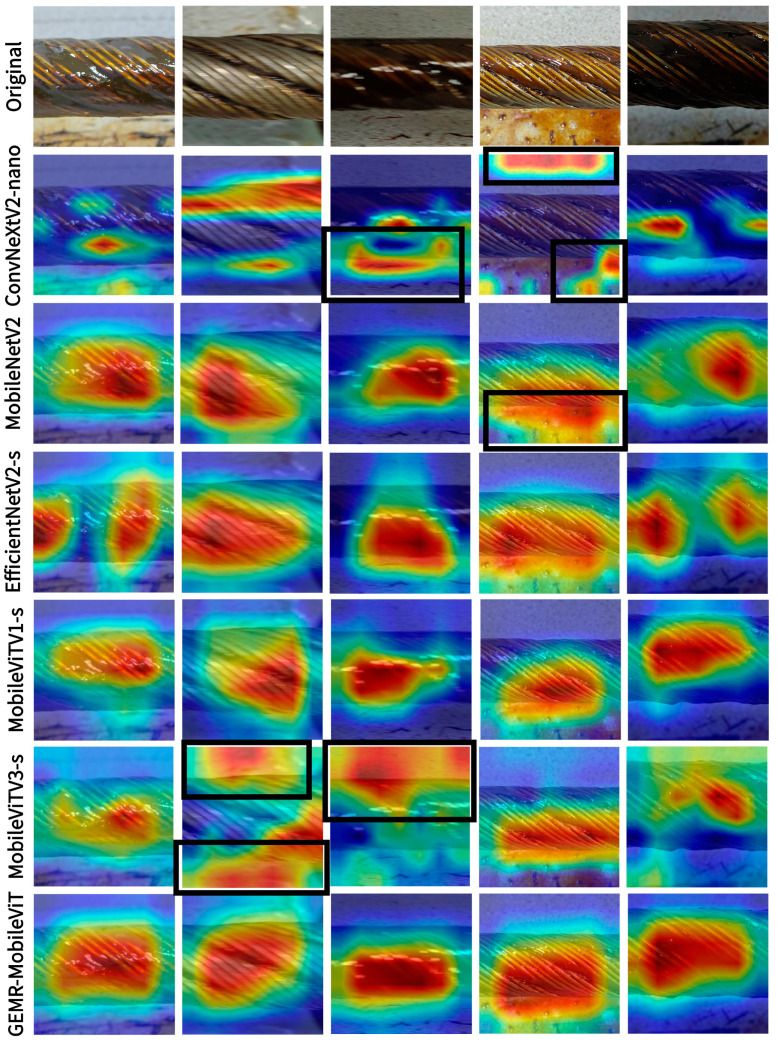
Images of different models visualized with Grad-CAM.

To better understand the regions of interest within the images that contribute to the model’s predictions, we used the Grad-CAM (Gradient-weighted Class Activation Mapping) method [27] to generate prediction heatmaps of multiple models for comparison, as shown in Figure 17. In this Figure, the red areas indicate the key feature regions that the models focus on, while the black boxes mark the wrongly attended parts of some models. It is evident from this Figure that different models have varying focuses on the target features in the image. Some models are easily interfered with by the background, such as the residual grease on the ground and the floor texture. As a result, they pay attention to incorrect background positions and miss the main area of the steel wire rope. Moreover, some models only extract features from individual small-scale areas on the steel wire rope surface. For example, they may only focus on the areas with more grease and darker colors in the grooves of the steel wire rope, or only on the brighter parts of the steel wires on the rope surface. Failing to capture the overall image features of the steel wire rope surface may lead to errors in the model’s classification results.

Through a comparison of the heatmaps of the different models, it can be found that the proposed GEMR-MobileViT has a broader focus on the entire surface area of the steel wire rope. In particular, compared with the pre-improved baseline model MobileViTv1-s, the area of focus on the feature parts of the steel wire rope surface by this model increases significantly. This can be explained by the fact that the introduction of the EMA mechanism enables its cross-spatial learning ability to integrate information from different spatial positions, strengthening the association between image regions. Meanwhile, the combined effect of the EMA mechanism and the transformer structure effectively enhances the model’s global modeling ability, allowing it to capture long-range feature associations in the image more efficiently. GEMR-MobileViT can not only capture local and subtle texture details but can also focus on and identify the overall area of the target within a relatively wide range, capture features from multiple positions of the target, and suppress background noise interference at the same time, thus achieving a more accurate and complete focusing and classification effect.

## 5. Discussion

This paper presents an improved GEMR-MobileViT model to address the challenge of the automated detection of grease thickness on wire rope surfaces. GEMR-MobileViT reaches a recognition accuracy of 96.63% on a self-constructed dataset, and it demonstrates significant advantages in terms of lightweight design. Its robustness across various lighting conditions and dynamic scenarios was also validated, thus providing crucial technical support for the automation of wire rope maintenance operations.

Throughout the research process, we encountered several significant challenges. First, in the setting of training hyperparameters, after multiple comparative experiments, the Adam optimizer was selected. It combines the methods of momentum and adaptive learning rate, enabling faster convergence. Compared with other optimizers, such as SGD, Adam can better handle sparse gradients and unstable gradient changes, and the accuracy is comparable. The initial learning rate was set to 0.0001, which is the optimal value obtained through experimental verification, this value can accelerate the training process while ensuring the convergence of the model. The commonly used cross-entropy loss function was chosen as the loss function, and the number of training epochs was set to 50; this choice was determined based on the model’s performance on the validation set to avoid overfitting caused by excessive training epochs. The batch size was determined according to the computer’s hardware performance and was set to 16 to ensure the stability of the model and the training speed.

Next, the inherent complexities of field image capture—such as motion blur, uneven lighting, and contaminants adhering to lubricated surfaces—created substantial difficulties for feature extraction. These challenges necessitated sophisticated preprocessing and robust attention mechanisms. To address these issues, we developed an EMA mechanism module alongside a residual connection structure to enhance the model’s capacity for feature extraction and fusion.

Moreover, the complex environment of wire rope lubrication operations presented another obstacle: ensuring the model’s generalization capability across varying operational conditions. This was addressed through the application of transfer learning strategies and comprehensive data augmentation techniques to bolster the model’s generalizability.

Additionally, the model continues to face prediction errors when differentiating between wire rope categories with very similar thicknesses. This fine-grained differentiation remains a notable challenge in object classification using visual methods, especially when visual distinctions between categories are minimal. Such scenarios make it difficult for the model to capture sufficient discriminative features for accurate classification. Therefore, further enhancing the model’s ability to discern fine-grained features, particularly when distinguishing between closely resembling categories, remains a critical area for ongoing development and research.

Future research may explore several key directions: First, expanding the dataset to encompass more extreme environmental conditions—such as marine environments, mining sites, and high-altitude cableways—along with various specifications of steel wire ropes and lubricating greases is necessary to enhance the model’s robustness. Second, investigating more efficient attention mechanisms, network architectures, and training processes could improve the model’s ability to discern fine-grained features among similar thickness categories. Furthermore, integrating the model with image segmentation networks, such as U-Net, may facilitate assessments of the uniformity of the grease distribution on wire rope surfaces. Finally, establishing a closed-loop lubrication system through the integration of a feedback control mechanism based on model predictions has the potential to enhance the autonomy of industrial maintenance workflows. Our study establishes a foundational framework for intelligent lubrication monitoring, and the proposed methodologies could be applicable to similar tasks in other industrial surface inspection domains.

Furthermore, the findings of this study offer a novel perspective for investigating the impact of lubricating grease on the tribological properties of steel wire ropes. The research by Feng et al. [28] demonstrated a direct correlation between the rheological properties of lubricating greases and the friction coefficients of steel wires, while Dyson et al. [29] highlighted the significant role of lubricants in reducing wear during the breaking-in phase of wire ropes. Future research could leverage our model to further explore the tribological behavior of steel wire ropes under varying thicknesses of surface lubricating grease. This could provide valuable insights into the fundamental relationship between lubrication and friction.

## 6. Conclusions

In this study, we developed an improved MobileViT model, named GEMR-MobileViT, for classifying and identifying grease thickness on wire rope surfaces. The model was trained and evaluated using a self-constructed dataset acquired from lubrication experiments conducted with a self-developed wire rope lubrication device. The dataset comprises five categories of grease thickness, providing a comprehensive basis for model training and validation. The following conclusions are drawn from the experimental results:

1. GEMR-MobileViT effectively addresses the challenges associated with recognizing grease thickness on wire rope surfaces. It achieves a recognition accuracy of 96.63%, representing a 4.4% improvement over the baseline MobileViT model. Additionally, the model exhibits a reduced computational complexity and parameter count, with 4.19 M params and 1.31 G FLOPs, corresponding to reductions of 15.2% and 10.3%, respectively, compared to the baseline. These improvements enable the model to offer lightweight advantages, facilitating the real-time and accurate identification of grease thickness, thereby advancing the intelligent automation of wire rope maintenance.
2. The proposed improvements to the MobileViT block, incorporating the EMA mechanism and residual connections, were experimentally validated to significantly enhance model performance. The integration of the EMA module within the local representation part of the MobileViT block forms an effective attention cascade with the transformer in the global representation part. Furthermore, the addition of a residual connection after the EMA module creates a synergistic effect, collectively strengthening the model’s feature extraction capabilities and effectively improving recognition accuracy in changing lighting and dynamic complex scenes.
3. GEMR-MobileViT exhibits strong robustness across various imaging conditions. Specifically, it achieves recognition accuracy rates of 95.38%, 93.33%, 100%, 95.83%, and 100% for the five grease thickness categories, respectively. Under different lighting conditions—high, medium, and low—the model’s recognition accuracy rates are 100%, 94.96%, and 100%, respectively. Additionally, in clear and blurred imaging scenarios, the model maintains high accuracy rates of 96.8% and 97.06%, respectively. These results demonstrate the model’s capability for the real-time recognition of dynamic grease thickness on wire rope surfaces under a wide range of conditions, thereby fulfilling the practical requirements for wire rope maintenance.
4. A comparative analysis with several mainstream models revealed that the GEMR-MobileViT model achieves the highest accuracy in classifying grease thickness on wire rope surfaces. It also exhibits significant advantages in terms of model parameters and computational complexity, making it highly suitable for deployment on embedded systems and mobile devices. This low-cost and efficient solution holds potential for integration into mobile wire rope lubrication devices, enabling real-time recording and feedback control. Future work will focus on deploying the model on such devices to create an intelligent system for wire rope maintenance, further advancing the development of automated and intelligent maintenance equipment.

## Figures and Tables

**Table 1:** Ranges of five categories of grease thickness.

Class	Unlubricated	Thin	Proper	Thick	Too Thick
Grease thickness (mm)	0	0–0.3	0.3–0.5	0.5–0.7	0.7 and above

**Table 2:** Number of images in each category before and after data augmentation.

Class	Unlubricated	Thin	Proper	Thick	Too Thick
Original samples	648	287	397	229	313
Total samples after augmentation	858	639	701	616	664

**Table 3:** Comparison of different models.

Model	Accuracy	F1-Score	Params (M)	FLOPs (G)	Model Size (MB)	*FPS* (f·S^−1^)
MobileNetV4-ConvSmall	91.71	92.15	2.4	0.25	9.63	121.51
MobileNetV2	90.16	90.53	2.2	0.33	8.64	118.86
EfficientNetV1-B0	87.56	88.07	4	0.41	15.47	116.32
ConvNeXtV2-femto	90.67	90.61	4.8	0.78	18.5	110.89
EdgeNeXt-small	84.46	84.39	5.3	0.96	20.15	110.45
MobileViT-xxs	90.67	90.25	1	0.27	3.65	119.05
MobileViT-xs	91.88	91.51	1.9	0.74	7.41	113.4
MobileViT-s	92.23	92.33	4.9	1.46	18.9	109.26
GoogLeNet (Inception-V1)	93.26	93.04	9.9	1.51	38	98.93
ResNet-18	92.75	93.28	11.2	1.82	42.68	102.47
ConvNeXtV2-nano	93.3	93.47	15	2.44	57.17	96.27
EfficientNetV2-small	93.34	93.73	20.2	2.9	77.58	93.15
Swin Transformer-tiny	89.64	90.33	27.5	4.37	105.21	85.18
NextViT-small	94.02	93.86	30.7	5.79	117.43	84.95
ConvNeXtV1-base	74.61	74.23	87.6	15.35	334.06	81.12
ViT-B/16	89.64	90.17	85.7	16.86	327.31	83.37

**Table 4:** Ablation study.

Model	GhostConv	EMA	Residual Connections	Accuracy	F1	Params (M)	FLOPs (G)	Model Size (MB)	*FPS* (f·S^−1^)
1	-	-	-	92.23	92.33	4.94	1.46	18.90	109.26
2	✔	-	-	92.23	92.23	4.18	1.29	18.98	111.96
3	-	✔	-	94.82	94.64	4.94	1.48	18.91	109.32
4	-	-	✔	88.60	87.71	4.94	1.46	18.89	107.20
5	✔	✔	-	93.26	92.92	4.19	1.31	18.99	108.57
6	✔	-	✔	91.19	90.82	4.18	1.29	18.98	111.85
7	-	✔	✔	95.34	95.25	4.94	1.48	18.91	110.97
8	✔	✔	✔	96.37	96.35	4.19	1.31	18.99	111.76

**Table 5:** Transfer learning results.

Transfer Learning	Accuracy	Accuracy—Mean	F1-Score	F1-Score-Mean	Total Training Time (min)
-	96.37	96.37	96.35	96.35	54.57
Method 1	96.89	96.63	96.66	96.39	34.41
Method 2	96.37		96.12		34.49

**Table 6:** Network position effects of EMA module.

EMA Position	Accuracy	F1-Score	Params (M)	FLOPs (G)	Model Size (MB)	*FPS* (f·S^−1^)
Local representation block	96.37	96.35	4.19	1.31	18.99	111.76
Global representation block	92.75	92.08	4.19	1.31	18.99	111.19

**Table 7:** Comparison of different attention mechanisms.

Attention Module	Accuracy	F1-Score	Params (M)	FLOPs (G)	Model Size (MB)	*FPS* (f·S^−1^)
SE	93.26	93.41	4.18	1.29	18.98	106.06
CA	93.26	93.24	4.18	1.29	18.99	111.88
CBAM	92.23	92.58	4.18	1.29	18.98	105.09
EMA	96.37	96.35	4.19	1.31	18.99	111.76

**Table 8:** Comparison of different models and GEMR-MobileViT.

Model	Accuracy	F1-Score	Params (M)	FLOPs (G)	Model Size (MB)	*FPS* (f·S^−1^)
MobileNetV4-ConvSmall	91.71	92.15	2.4	0.25	9.63	121.51
MobileNetV2	90.16	90.53	2.2	0.33	8.64	118.86
EfficientNetV1-B0	87.56	88.07	4	0.41	15.47	116.32
ConvNeXtV2-femto	90.67	90.61	4.8	0.78	18.5	110.89
EdgeNeXt-small	84.46	84.39	5.3	0.96	20.15	110.45
MobileViT-xxs	90.67	90.25	1	0.27	3.65	119.05
MobileViT-xs	91.88	91.51	1.9	0.74	7.41	113.4
MobileViT-s	92.23	92.33	4.9	1.46	18.9	109.26
GoogLeNet (Inception-V1)	93.26	93.04	9.9	1.51	38	98.93
ResNet-18	92.75	93.28	11.2	1.82	42.68	102.47
ConvNeXtV2-nano	93.3	93.47	15	2.44	57.17	96.27
EfficientNetV2-small	93.34	93.73	20.2	2.9	77.58	93.15
Swin Transformer-tiny	89.64	90.33	27.5	4.37	105.21	85.18
NextViT-small	94.02	93.86	30.7	5.79	117.43	84.95
ConvNeXtV1-base	74.61	74.23	87.6	15.35	334.06	81.12
ViT-B/16	89.64	90.17	85.7	16.86	327.31	83.37
MobileViTv3-s	95.34	95.14	3.69	1.26	14.14	110.78
GEMR-MobileViT-s	96.63	96.39	4.2	1.31	18.99	112.05

## Data Availability

The experimental results’ data are available upon reasonable request to the corresponding author. The steel wire rope image dataset used in this study is proprietary and cannot be shared due to confidentiality agreements.
